# Strengthening monitoring systems and inter-departmental convergence to drive program improvements

**DOI:** 10.1186/1753-6561-6-S5-O18

**Published:** 2012-09-28

**Authors:** George Philip, Ashok K Singh, Madhuri Narayanan, Ragini Pasricha

**Affiliations:** 1 IntraHealth International, Delhi, India

## Introduction

Under the National Rural Health Mission, an initiative has been taken to provide 15 integrated maternal, child health, and nutrition services through outreach sessions like the Village Health and Nutrition Days (VHND). Such sessions are organized in the village at a fixed day, place and time through convergent actions by the departments of health and the department of women and child development. Due to inadequate dissemination of guideline for conducting outreach sessions, health workers often lacked clarity in understanding their role. Services are often limited to immunization. There is inadequate monitoring of these sessions with no functional forum to review the performance of the session and problem solving by both the departments involved.

## Methods

The IntraHealth-led Vistaar Project provided technical assistance to the Government of Uttar Pradesh to expand the quality and coverage of VHNDs in eight districts. The project worked with district staff of the ministries of health and women and child development through:

• Orientation of district officials and frontline workers on VHND guidelines and creating role clarity;

• Promotion of structured monitoring through an observation checklist and orienting supervisors on its use;

• Designing an auto analysis package for regular analysis of observation and related capacity building to the system;

• Facilitation of joint micro-planning;

• Activation of convergence platforms to meet regularly, with VHND review on the agenda using data from monitoring visits to review program performance and drive improvements in access, coverage, and range of service; and

• Orienting members of *Panchayati Raj Institutions* and Village Health and Sanitation Committees on their role.

## Results

Results of the study show that the project intervention resulted in improved inter-departmental convergence. This is reflected in joint micro-planning, the presence of all the three front line health workers at VHND sessions to provide an expanded range of services and joint review of program performance.

Findings from the project management information system indicate that between July-September 2009 to April-June 2011, VHND sessions at which frontline workers from the both the government departments were present, increased from 61% to 81%, the mean number of services offered during VHND increased from 5.6 to 8.1 and blocks where monthly convergence meetings were held, increased from 58% to 94%.

In terms of improved monitoring, we observed that percentage of VHND sessions at which supervisors from both the departments of health and women and child development were present, increased from 21% to 26%. Observations by supervisors resulted in immediate feedback to frontline workers.

These results are consistent with end line survey (Jan-Mar 2012) findings. Additionally, end line data showed that among currently pregnant women, awareness of VHND was widespread (88%) and they knew both the date and location of VHNDs in their area (67%). More than three quarters of pregnant women reported presence of all the front line workers at the VHND site.

## Discussion

Improving monitoring of VHNDs was critical at two levels. At the field level, it resulted in feedback for improving the quality of services. At the managerial level, the review of data from monitoring visits resulted in improved worker deployment and ensuring equipment and supplies to provide an expanded range of services.

Increasing ownership through orientation on guidelines and joint planning and using data during regular review meetings create opportunities for successful program delivery. While data are collected for most government programs, it is passed on to the state and national level and rarely used locally to assess and improve program performance. In order for data to be used at the block and district, capacity building was required for collection, verification, randomization and analysis. The Project had maintained the MIS throughout the life of the Project and in its sustainability phase it has initiated capacity building at the block and district levels.

Another learning is that if just top level indicators are tracked, it does not serve the data needs of program implementers who may require more complete information on which gaps to address. A comprehensive checklist is more useful at the district and block levels. VHND is a priority under the National Rural Health Mission in India and the lessons learned about convergence, improved monitoring and use of data has potential for replication and wide scale-up.

## Competing interests

Authors declare that they have no conflict of interest.

## Funding statement

This study was funded by the United States Agency for International Development Cooperative Agreement 386-A-00-06-00162-00.

